# England's new Mental Health Act represents law catching up with science: a commentary on Peter Lepping's ethical analysis of the new mental health legislation in England and Wales

**DOI:** 10.1186/1747-5341-2-16

**Published:** 2007-08-06

**Authors:** Anthony Maden

**Affiliations:** 1 The Paddock Centre, Crowthorne, Berks RG45 7EG, UK

## Commentary

Those of us working in mental health tend to forget that much of medical ethics consists of coming to terms with scientific discoveries. Whilst society is still digesting the news that we can choose a child's sex, genome researchers tell us we will soon be able to know its medical future as well. In this world of breakthroughs and bombshells, psychiatry is the hick town where nothing happens; its dilemmas are the timeless ones. Ethicists have always worried about balancing the rights of the individual against those of the general public, and the antiquarian overtones of terms such as non-maleficience remind us that Hippocrates (460–370 BC) got there first. So Lepping's paper has an elegiac tone; he laments the passing of respect for individual rights, swept away by the tide of utilitarianism.

This approach is essentially nostalgic and fails to do justice to progress in mental health care. Lepping attributes his premises, including "the failure of community care, the need for new legislation and new community treatment realities", to the Department of Health in 1998 [1] But it is only possible to understand these concerns if one looks a little further back at their context, and 1983 is the logical place to start because that was when our present Mental Health Act came into force.

A few years later, psychiatry discovered violence. Doctors in 1983 believed there was no link between mental illness and violence [2] but they were proven wrong. A significant positive association between schizophrenia and violence is now well established [3-5] and services have had to adapt to a risk that was unknown 25 years ago.

As ethical concerns go, the association of violence with mental illness – and, in some cases, with inadequate medical treatment – is a Big One. It is extremely rare for medical treatment ever to present a risk to a third party so, when it does present such a risk, we should not be surprised that the public finds it unacceptable. The general principle is that individuals readily tolerate risks for which they volunteer, and when they are counterbalanced by pleasure or other benefit; they do not tolerate risks imposed upon them without their consent, particularly when there is no counterbalancing benefit.

Homicides by the mentally ill account for 5–10% of all killings in England and Wales. Whilst they are not increasing [6] neither are they falling, and recent research shows that non-compliance with treatment in the community is an important cause of such tragedies [7]. About half of all patients with schizophrenia discontinue medication within twelve months, and discontinuance is more likely in patients with other risk factors for violence, including personality disorder and substance misuse. The best that professionals can do under the 1983 law is sometimes not enough to prevent foreseeable disaster [8]. These facts ought to be included in an ethical analysis of the proposed new community treatment order, and similar concerns troubled our Dept of Health throughout the 1990s.

The ethical debate was crystallised by the case of the psychotic patient who killed Jonathan Zito in London in 1992. The Inquiry [9] into the incident revealed inadequate care during the preceding four years of involvement with mental health services, and it led to major reforms of the way in which care is delivered [10]. In fact, the best way to understand the new Mental Health Bill is as a further stage in the process that began with the death of Jonathan Zito.

We should also bear in mind that the same time period has coincided with a general increase in aversion to risk which is not confined to mental health or, indeed, to any one country. There is no ethical basis for arguing that mental health should remain exempt from this trend, which has swept through most democracies. Nor has mental health been singled out for special treatment; recent criminal justice legislation in England and Wales has resulted in judges imposing about 150 indeterminate sentences each month on the grounds of public protection.

Despite medical ignorance about the link between mental disorder and violence at that time, the 1983 Act was actually based on notions of risk management. It restricted the use of detention to situations in which it was necessary for the health or safety of the patient, or for the safety of others. Later scientific developments tend to support this approach so it is reasonable for the Government to persist with risk-based legislation.

That is not to say that the Government is right. However, ethical advocates of a change to capacity-based legislation are under an obligation to deal with the science. "Why should separate statutes govern the involuntary treatment of "physical" and "mental" illness?", ask proponents of capacity-based legislation [11], whilst refusing to consider the obvious answer: "Because mental illnesses are associated with a risk of violence in a way that is never encountered in physical illness".

Replacement of the "treatability test" in psychopathic disorder by a requirement for all mental disorders that "appropriate treatment is available" also reflects scientific progress. Psychopathic disorder in 1983 was poorly defined and overlapped with criminality to such an extent it was impossible to tell the difference. The Psychopathy Checklist [12] now gives diagnostic reliability comparable to that in schizophrenia and identifies a condition, found in less than 10% of prisoners, which correlates with recidivism and violence risk [13]. Outside forensic settings, in the MacArthur study of ordinary psychiatric patients, a psychopathy score was the best single indicator of violence risk [14]; it is becoming an indispensable concept in mental health care rather than the dubious, marginal diagnosis it was when the 1983 Act was drafted.

Treatment of personality disorder has also improved. Cognitive behavioural interventions reduce re-offending risk in violent and sexual offenders, many with serious personality disorders, achieving effect sizes comparable to treatments such as heart surgery for angina, or AZT for Aids [15]. The consensus of medical opinion remains that compulsory treatment is appropriate for only a minority of patients, whether the diagnosis is personality disorder or schizophrenia, but these advances in diagnosis and treatment have removed the rationale for a legal distinction between these conditions.

The third major change may be the most significant in the longer term, by allowing staff of other disciplines to take on the role of clinical supervisor for patients receiving compulsory treatment. The Government is right to ignore mutterings of medical discontent, which are inevitable as doctors feel their privileged position is threatened. Mental health care has become truly multidisciplinary since 1983, and doctors do not have a monopoly on ethics or (with few exceptions) technical skills. Patients' needs go far beyond the medical and other disciplines will sometimes do a better job as clinical supervisors; that basic fact is all that matters. The main safeguards will concern medication, given its importance in managing the risks associated with mental illness, and it will be essential to ensure that a non-medical clinical supervisor is no less assertive in this respect.

Given adequate training and support, these legal changes (the Act received Royal assent on 20^th ^July 2007) could greatly improve the treatment of that minority of patients for whom compulsion is necessary. They may also leave mental health services looking very different when mental health legislation is next reviewed.
